# Endophyte Infected Tall Fescue: Plant Symbiosis to Animal Toxicosis

**DOI:** 10.3389/fvets.2021.774287

**Published:** 2021-12-24

**Authors:** Taylor D. Ferguson, Eric S. Vanzant, Kyle R. McLeod

**Affiliations:** Ruminant Nutrition Laboratory, Department of Animal and Food Sciences, University of Kentucky, Lexington, KY, United States

**Keywords:** ruminant, bovine, tall fescue, endophyte, ergot alkaloids

## Abstract

Endophyte-infected fescue is a major cool season forage used for livestock production in the United States and through other areas of the world. A unique aspect of this forage resource is the symbiotic relationship with an endophytic fungus (*Epichloë coenophiala*) that has detrimental impact on herbivores due to toxic ergot alkaloids. Research over the past 50 years has unveiled details regarding this symbiotic relationship. This review focuses on the origin of tall fescue in the United States and the consequences of its wide-spread utilization as a livestock forage, along with the discovery and toxicodynamics of ergot alkaloids produced by *E. coenophiala*. The majority of past ergot alkaloid research has focused on observing phenotypic changes that occur in livestock affected by ergot alkaloids, but recent investigation of the metabolome, transcriptome, and proteome have shown that fescue toxicity-related illnesses are much more complex than previous research suggests.

## Introduction

One of the most wide-spread and heavily utilized forages in the United States is the cool-season grass, tall fescue (*Festuca arundinacea* Schreb.). To some extent, all sectors of the livestock and equine industries rely on tall fescue for grazing, but beef cow-calf operations became especially dependent on the grass after its widespread introduction to the country during the mid-twentieth century (1). The species appeared to be well-suited for livestock grazing because of its hardiness, nutritive qualities, and ease of cultivation, however, consumption of tall fescue quickly became associated with poor animal health and performance (2). The multifaceted syndrome, dubbed tall fescue toxicosis, can be costly to producers and has garnered attention from scientists for many years. Once it was recognized that the disease was caused by ergot alkaloids, which are present in most tall fescue through its relationship with the endophytic fungus *Epichloë coenophiala*, research began to focus on how alkaloid consumption altered body homeostasis in order to fully understand the underlying mechanisms of fescue toxicosis (3). There is still much to be discovered regarding the influence of endophytic alkaloids on essential metabolic pathways, such as protein expression, insulin signaling, and lipid metabolism, as their effects are virtually unknown at the present time. This review provides a comprehensive examination of the agronomic properties of endophyte-infected fescue and its symbiotic relationship, with a particular focus on the absorption and metabolism of associated alkaloids, including their impact on cattle performance and health. Additionally, the latest research showing the impact of fescue derived alkaloids on the metabolome, transcriptome, and proteome will be discussed.

## History of Tall Fescue and Fescue Toxicosis

### Introduction and Selection in the United States

Even though tall fescue is the most important pasture grass in the United States, its introduction into the country is shrouded in speculation. The story likely begins with another native of Europe and close relative, meadow fescue (*Festuca pratensis* Huds.), which was first introduced into cultivation around 1820, but has long been recognized for its value as a forage plant (4). Almost all the meadow fescue seed planted in the US until the late 1880s was imported from England, tall fescue was likely a contaminant in that seed (5). These accidental additions to meadow fescue pastures were quickly recognized for their ability to thrive even in conditions where meadow fescue began to wane. By 1900 it was proving its worth for pasture and mowing in grass trials in Kentucky (6) and Virginia (7); and was praised for its superior growth, height, competitive ability, and drought tolerance.

The popularity of tall fescue was not fully realized until the release of two cultivars in the 1940s, Alta and Kentucky 31 (KY-31). Starting in 1918, the ecotype Alta was selected for winter hardiness, persistence, and ability to remain green during the dry summers of western Oregon; and then released cooperatively by the Oregon Agricultural Experiment Station and the USDA (8). Alta was planted throughout the Pacific Northwest and intermountain regions of the western United States. The ecotype, KY-31 was first collected in 1931 from a mountain pasture in Menifee County, Kentucky. The population had been under natural selection on the site when the property was purchased in 1875 and local farmers held it in high regard for pasture and erosion control (9). The Kentucky Agricultural Experiment Station conducted lengthy tests of the cultivar, and it was released in 1942. KY-31 was noted for its dependability, adaptability to a wide range of soils, and ability to provide grazing over much of the year (1). With the release of KY-31, the popularity of tall fescue soon eclipsed that of other cool season perennial grasses, mostly because of its greater persistence in pastures compared to other species. These qualities can be attributed to its course, deep root system which develops a thick sod; allowing it to grow on sites with moist, heavy soils, that are often waterlogged or flooded, making tall fescue particularly valuable for conservation purposes (1). The previously barren and brown winter landscape of the southern United States was transformed during the 1940s and 1950s by the widespread planting of tall fescue for forage, soil conservation, roadside cover, and turf (3).

During the 30 years following the release of Alta and KY-31, tall fescue cultivation rapidly spread in the United States, until it was the predominant cool-season perennial grass. In 1940 it grew on ~16,000 ha, over 1.6 million ha in 1956, and by 1973 it occupied an estimated 12–14 million ha; the species also spread from its original range in the transition zone and is now grown from Florida to Canada in a wide range of climatic conditions (1, 8).

### Tall Fescue Toxicity

With the widespread use of tall fescue for pasture came the rise of a conglomerate of health issues that were discovered to be a direct result of livestock consuming the grass. By the 1950s, fescue had gained a reputation for causing problems in animals that resulted in poor performance (10). This puzzled animal scientists since well-managed fescue was a high-quality forage when it came to crude protein, digestible dry matter, amino acid, and mineral content, which should have resulted in acceptable animal performance (2). The first health issue to be reported and associated with fescue pastures was the syndrome fescue foot. In the summer months, animals consuming large amounts of tall fescue struggle to dissipate heat, resulting in excessive panting, and spending extended time in shade or in ponds. In the winter, animals cannot maintain peripheral body temperature, which can result in frost bite and subsequent loss of ears, tail switches, and hooves (11). This was first reported in Australia by Cunningham (12) and then in New Zealand by Pulsford (13), who said it had been known by stockman for many years. In the United States the first report came from Colorado in 1952; it had been recognized for about 30 years, but was confused with other conditions like foot rot, frozen feet, or ergot poisoning (14).

The second syndrome, known as fat necrosis, occurs where high rates of nitrogen fertilizer are applied to tall fescue pasture; mainly from poultry litter or other manure. Hard fat accumulates in the abdominal cavity of cattle, which do not show symptoms until vital body processes are affected. Most frequently digestive upset occurs when the necrotic tissue constricts the intestines (15). Fat necrosis may also interfere with the functions of the kidney and heart, as well as cause difficult births if the birth canal is surrounded (16, 17). This problem was first clinically diagnosed by Williams et al. in the United States in 1967 (17).

The third syndrome, first described by Robbins in 1973 is commonly called summer slump, because it is exhibited especially during the summer, when temperatures are highest (18). Symptoms are varied and include nervousness, elevated body temperature, intolerance to heat, failure to shed winter coat, depressed feed intake, poor weight gains and milk production, excess salivation, and reduced conception rates (19). The major economic losses to the United States beef industry associated with consumption of endophyte infected tall fescue can mostly be attributed to this disorder (20). Poor performance has also been observed in other livestock. Dairy cattle grazed on tall fescue have lower milk production than on other grasses (21) and mares have higher foal mortality rates and agalactia (22).

Even though financial losses attributed to tall fescue are great, producers often do not recognize the problem, given that the signs can go undetected until they become severe, this makes fescue toxicosis the largest animal health related production cost in the grazing industry (23). A survey in 1993 of state extension specialists where tall fescue is most common, showed that losses from reduced conception rates and weaning weights in beef cattle total $609 million annually (24). Profitability of beef stocker cattle grazing on tall fescue is also compromised because of low daily gains. Losses to the horse industry from fescue toxicosis are exceedingly difficult to estimate, but nearly all the states reported reproduction issues. Compared to those of cattle, increased foal mortality may not result in as many deaths, but considering the price of horses, especially in the thoroughbred industry, these losses still hold a high monetary value on an individual basis. When regarded in today's dollars and considering the impact on the equine and small ruminant industries, the combined annual loss to the livestock industry from fescue toxicosis likely exceeds $1 billion (23). The cost of this disease drove researchers to attempt to uncover the root of these syndromes.

### Determining the Cause of Fescue Toxicity

During the 1950s−1970s, research erroneously concentrated on possible culprits such as external plant fungi, plant alkaloids, anions, and toxins produced in the rumen. This delayed any breakthroughs in determining the cause of fescue toxicity (15). Dissection of the limbs of cattle experiencing lameness while consuming tall fescue revealed that the toxic agent in the grass caused vasoconstriction and thrombosis of the arteries, which obstructed blood flow. This, plus low environmental temperatures were determined to cause the gangrene of the extremities associated with fescue foot (25). Chemical tests isolated several alkaloids from toxic tall fescue that were similar to those found in the ergot fungus, *Claviceps purpurea*, but no ergot sclerotia from that fungus were detected by visual examination (26, 27). Discovering ergot alkaloids in fescue was very important, but these early reports were mostly ignored by scientists working on fescue toxicosis at the time. Until a suitable bioassay could be perfected, progress in isolating the toxic component was very slow. The syndrome was experimentally produced in cattle by Jacobson et al. (11), using an extract chemically fractioned from toxic forage; this method allowed them to define the physiological effects of tall fescue consumption. They also demonstrated that poor circulation resulting in gangrene of the extremities was not caused by an abrupt halt in blood flow, since gangrene only became apparent after about 10 days. Their findings clearly indicated that there was vasoconstriction, but the specific causes were still unknown.

Progress began in 1973, when J. D. Robbins, J. K. Porter, and C. W. Bacon focused their research on a toxic tall fescue pasture in northern Georgia. From that single pasture, this group of scientists was able to isolate several species of fungi, which served as the basis for a multitude of grass toxicity studies. Three species were isolated from this pasture, all belonging to the tribe Balansiae: *Balansia epichloe* (Weese) Diehl, *B. henningsiana* Moell, and *Myriogensopora atrementosa* (Berk. and Curt.) Diehl. Unlike their saprophytic or pathogenic relatives, these species are unique because they are endophytic, and not virulent to their grass hosts. The species of *Balansia* primarily associate with warm season weed and rangeland grass species, such as bentgrass, bluestem, crowngrass, and others, which are commonly found in pastures of tall fescue and contribute to the toxicity of tall fescue during the summer months (28).

The most important series of toxicological studies during this time were conducted primarily by J. K. Porter. He was able to demonstrate that these endophytic species of fungi were toxic and could potentially synthesize ergot alkaloids. Fungal spores were collected and allowed to germinate, then cultures were incubated in appropriate medium so alkaloids could be produced *in vitro*. Inocula prepared from these cultures, containing indole compounds, proved to be toxic to chicken embryos (29). Chemical extraction isolated four clavine-type alkaloids from cultures of *B. epichloe*: chanoclavine (I), agroclavine, penniclavine and elymoclavine, which are commonly found in species of *Claviceps*. Chanoclavine (I) was also produced by *B. claviceps, B. henningsiana* and *B. strangulans*. The lysergic acid derivatives, ergonovine and ergonovinine, were isolated from *B. claviceps* and *B. epichloe* (30–32); this was the first demonstration of these compounds in fungi outside the genus *Claviceps*. These findings suggested that ergot alkaloids produced by these species may be involved in cattle toxicity syndromes where species of *Claviceps* are absent.

After the discovery of endophytic *Balansia* species in warm season grasses and the demonstration that biosynthesis of ergot alkaloids was limited to the family Clavicipitaceae, tribe Balansiae, this group of researchers began searching for a species of *Balansia* or similar endophyte in the toxic tall fescue in the pasture in north Georgia. A 2-year observation of the pasture grazed by cattle suffering fescue toxicity symptoms, did not reveal a specific *Balansia* taxon in the grass, but they did isolate another fungal endophyte, which at the time was believed to be *Epichloë typhina* (33). The cattle suffering toxicosis grazed pastures with infection rates of 100%, while pastures with cattle in good condition had infection rates of 0–50%. The identity of the tall fescue endophyte, which was symptomless and non-sporulating, was originally reported to be *E. typhina* based on the work of Sampson (34). This was challenged later by Morgan-Jones and Gams (35), who found the endophyte to be an anamorph of *E. typhina* and not identical. To classify the anamorphs of *Epichloë*, they created the section Albo-lanosa within the genus *Acremonium* and named the endophyte *A. coenophialum*. In 1996, a phylogenic examination of species in *Acremonium* determined that the anamorphic tall fescue species was classified inappropriately and proposed the monophyletic genus *Neotyphodium* (36). The species was reclassified again in 2014, when a nomenclatural ruling called for *Epichloë* and its anamorphs in *Neotyphodium* to be placed into one genus (37). Thus, the tall fescue endophyte is currently named *Epichloë coenophiala*. Further evidence from grazing trials confirmed that this fungal endophyte was the causal agent of fescue toxicosis.

A 3-year trial in central Alabama showed that steers in paddocks with 18% *E. coenophiala* infestation, had 51% higher average daily gains (ADG) than steers in paddocks with 80% infestation (38). These findings were confirmed by two more trials in the following years. One demonstrated again that steers grazing fescue with low infestation rates would have higher gains. In this case, steers in paddocks with 5% infestation, had 66% greater ADG and 28% greater gain per acre than animals in paddocks with 94% infestation (19). Another produced similar results by feeding steers fungus-free and infested KY-31 hay and seed; the presence of the fungus in both the hay and the seed diets decreased daily gains and feed intake (39). Steers eating infested grass, hay, or seed, showed typical signs of toxicosis; elevated body temperature, rough coat that did not shed, excessive salivation, and nervousness; while steers on low-endophyte grass or fungus-free feed were in excellent condition, tolerated heat, and showed no signs of nervousness (19, 39). The association of endophyte infection with tall fescue toxicosis in cattle (40, 41), sheep (42), and horses (43, 44), was demonstrated by several others in the following years, further confirming that endophyte-infected tall fescue is toxic to livestock.

Even though ergot alkaloids were produced in culture from the tall fescue endophyte in 1979, the toxins were not detected in plants until 1985. Using tandem mass spectrometry, significant levels of ergopeptine alkaloids were detected in extracts from tall fescue pastures with recent histories of toxicity in cattle. Ergot alkaloids were present in all aboveground parts of the plant, with 10–50% being ergopeptines. Specifically, ergovaline, ergosine, ergonine, ergoptine, and ergocornine were reported, with the predominant type being ergovaline at 84–97% of the total ergopeptine fraction (45). Small amounts of ergotamine have also been found in endophyte infected fescue (46). Historically, ergovaline has been recognized as the major ergopeptine alkaloid produced by the endophyte, and the primary cause of reduced plasma prolactin and vasoconstriction (47). However, it is unwise to overlook the other ergopeptine alkaloids and metabolites found in infected tall fescue. In the past it has been difficult to study differences between specific alkaloids due to the lack of commercially available ergovaline.

A survey of seed and pasture from all over the United States was conducted in 1987 to detect potentially damaging levels of endophyte. They found that about 95% of tall fescue pastures showed varying degrees of endophyte infection. This widespread occurrence was largely due to the overwhelming acceptance of KY-31 from its release in 1943 into the 1960s; this cultivar also had a higher level of infection than most of the others surveyed (48). The species was widely dispersed in the eastern part of the country and during this time the forage industry was becoming more aware of the losses sustained when cattle grazed on infected fescue. At the time it was thought that the solution was to remove the endophyte from tall fescue altogether.

### Failure of Endophyte-Free Cultivars

As researchers studied grass endophytes, they realized they were sensitive to storage conditions. When tall fescue seed was exposed to hot, humid conditions during storage, the endophyte would die, but the seed remained viable. In the past, the high demand for KY-31 seed meant that most of what was harvested in the summer was sold and planted within months, so the seed was not exposed to conditions sufficient to kill the toxic endophyte, which facilitated its spread. Now with a way to remove the endophyte, new cultivars of fungus free and non-toxic tall fescue were developed, the first being AU Triumph, which was released in 1982 (49). Thorough testing showed excellent animal performance on pastures of AU Triumph and farmers readily replanted toxic pastures with endophyte-free seed. Unfortunately, grazing practices were not altered for these E- pastures and within a few years, farmers noticed that unlike the famous KY-31, the new cultivar could not persist through drought and heavy grazing. Even pastures planted with other cultivars containing low levels of endophyte infestation (<25%) had decreased stand persistence during periods of stress compared to pastures that were highly infected (90+%). The loss of stand in the low-infested pastures indicated a competitive advantage for infected tall fescue (50). Selection for more persistent endophyte-free varieties was attempted but was unsuccessful, and it became apparent that agronomic performance was enhanced by a mutualistic relationship between tall fescue and the endophytic fungus. While endophytes had previously been considered parasitic, evidence that they conferred superior fitness to the host plant and that these benefits were multifaceted were piling up.

### Symbiotic Relationship

Tall fescue research up to this point had mostly focused on the detrimental effects the endophyte had on livestock production, so the biological significance of the fungus was not fully understood. This was quite apparent after the premature release of the E- tall fescue cultivars and their subsequent failure in stressful environments. The ultrastructural association between grass and fungus, as well as the growth and survival benefits granted to infected grasses, suggested that a symbiotic relationship formed early in their respective evolutions (51). *E. coenophiala* has a relatively simple life cycle. The species is asexual, so the only route of dissemination is by maternal transmission through host embryos. Fungal hyphae colonize seeds in inflorescences early in development, and occupy the embryonic axis, including the shoot atypical meristem and the scutellum (52). The endophyte lies dormant in the seeds until they are planted and germinate, or it dies in storage. Once germinated, elongated hyphal filaments colonize the extracellular spaces of leaf sheaths, meristems, and internodes of growing stems, but not roots, and absorb nutrients directly through the hyphal wall (52). In this relationship, the fungus benefits by receiving nutrients, protection, and a means for reproduction and dissemination, while the host plant receives a variety of advantages in overall vigor along with protections against biotic attack. Several ecological and physiological processes in the fescue plant were found to be influenced by symbiosis with the endophyte; but a single mechanism by which the endophyte promotes host fitness is not apparent (53).

Since its accidental introduction, tall fescue has been celebrated for stand persistence and tolerance of grazing during dry conditions, these are major benefits of endophyte infestation. The mechanisms enabling this ability are complex and not fully understood. During severe water deficit, endophyte infected plants showed enhanced tiller density and survival, which conferred population stability and proved advantageous in the following year (54). Greenhouse experiments showed that E+ plants were more productive than E- plants of the same cultivar at mild moisture stress (−0.05 MPa). During severe moisture stress (−0.5 MPa), 75% of the E- plants died, while all the E+ plants survived. Infected plants often showed greater shoot mass and tiller numbers than endophyte-free, and previously drought stressed infected plants also had much greater regrowth after harvest (55). This fitness helped tall fescue spread until it was one of the most important pasture grasses in the United States and prevail as a cool-season species in states south of its typical area of adaptation. The transition zone in the United States represents a progression, from north to south, of increasing summer water deficit caused by high evaporative demand and low soil water-holding capacity, such as the rocky soils in the Ozark Highlands and southern Appalachian Mountains. The endophyte helps tall fescue compete and survive in this transition zone when growing conditions favor other plant species (53).

Indirectly, the endophyte also enhances host persistence by deterring herbivory. The production of certain chemicals, including alkaloids, deters grazing animals, insects, and nematodes. This allows the plant to retain high leaf area and strong root systems, thus avoiding energy depletion and maintaining the ability to acquire water and nutrients in stressful conditions. Infected fescue gains resistance to herbivorous insects which would deplete the leaves (56) and nematodes which would destroy the root mass (57). These findings led to a greater understanding of the complex relationship between the endophyte and tall fescue and had monumental implications for cultivar development and pasture management.

### Success of Non-toxic Endophyte Cultivars

In the late 1980s, Joe Bouton listened to a talk given by Gary Latch, a researcher from New Zealand, about endophytic fungi infection in tall fescue's close relative, perennial ryegrass. Afterwards, while Bouton and Latch discussed their research, Latch mentioned that he had identified endophytes in perennial ryegrass that did not produce alkaloids toxic to livestock, which were the cause of ryegrass staggers in sheep. What made this discovery unique was that while the ryegrass endophyte didn't produce the toxic alkaloid lolitrem B, it still produced peramine, a deterrent against the main insect pest of perennial ryegrass in New Zealand, the Argentine stem weevil (58). This differed from endophyte-free cultivars which did not produce any protective alkaloids, reducing survivability and usefulness as a pasture grass. Bouton visited New Zealand several years later to work with Latch and his associates; together they isolated a naturally occurring, non-toxic strain of endophyte. The result was the AR542 endophyte, which does not produce ergot alkaloids, now marketed as MaxQ in the United States (59). The strategy was to kill the toxic endophyte in seed from the best available tall fescue cultivars, then reinfect seedlings with the non-ergot alkaloid producing endophyte, thus eliminating the toxic effects in livestock, while maintaining the symbiotic benefits between endophyte and grass. The main commercial “novel” product on the market is Jesup MaxQ, the result of reinfecting the tall fescue cultivar Jesup. This novel cultivar demonstrated stand survival equal to toxic E+ tall fescue, and decidedly better than E- fescue (59). Jesup MaxQ also did not express the effects of ergot alkaloids on grazing animals, so avoided the negative effects on animal performance associated with E+ pasture (59–61). To date, this cultivar has produced no animal toxicity problems. Currently, the most dependable method for eliminating animal losses from tall fescue toxicosis is replacing toxic tall fescue pastures with novel cultivars. Replacement of pastures has a substantial up-front cost, but improvement in animal production can generously repay the investment.

## Ergot Alkaloids and Animal Toxicodynamics

### Alkaloids in Medicine

Alkaloids are organic, N-containing, basic compounds, which are naturally produced by a variety of organisms, most often plants. Many plant-derived alkaloids are familiar, as they have important medical uses, such as morphine (analgesic), quinine (antipyretic/antimalarial), atropine (anticholinergic), and vincristine (antitumor). Others are addictive like cocaine, heroin, nicotine, and caffeine; or very toxic like strychnine and coniine. The multitude of ergot alkaloids were named for the ergot fungus *Claviceps purpurea*, their first known producer and cause of several epidemics during the middle ages. Ergot alkaloids all contain a tetracyclic ergoline ring system or a biosynthetic precursor; enzymatic modification of the ergoline ring results in a plethora of bioactive natural products that can be used as pharmaceutical treatments for a variety of ailments (62). They have a strong affinity for serotonin, dopamine and adrenergic receptors because of structural similarities with neurotransmitters, so they can be potent drugs; for example, methylergometrine is used to stop bleeding after childbirth, ergotamine to treat migraines, and bromocriptine is used to treat Parkinson's disease (62). Those that have been isolated from grass infected with the endophyte *Epichloë coenophiala* are considered the causal agents of animal disorders associated with tall fescue consumption and include clavines, lysergic acid, simple lysergic acid amides, and ergopeptines (63).

### Synthesis and Structure

**Figure 1:**
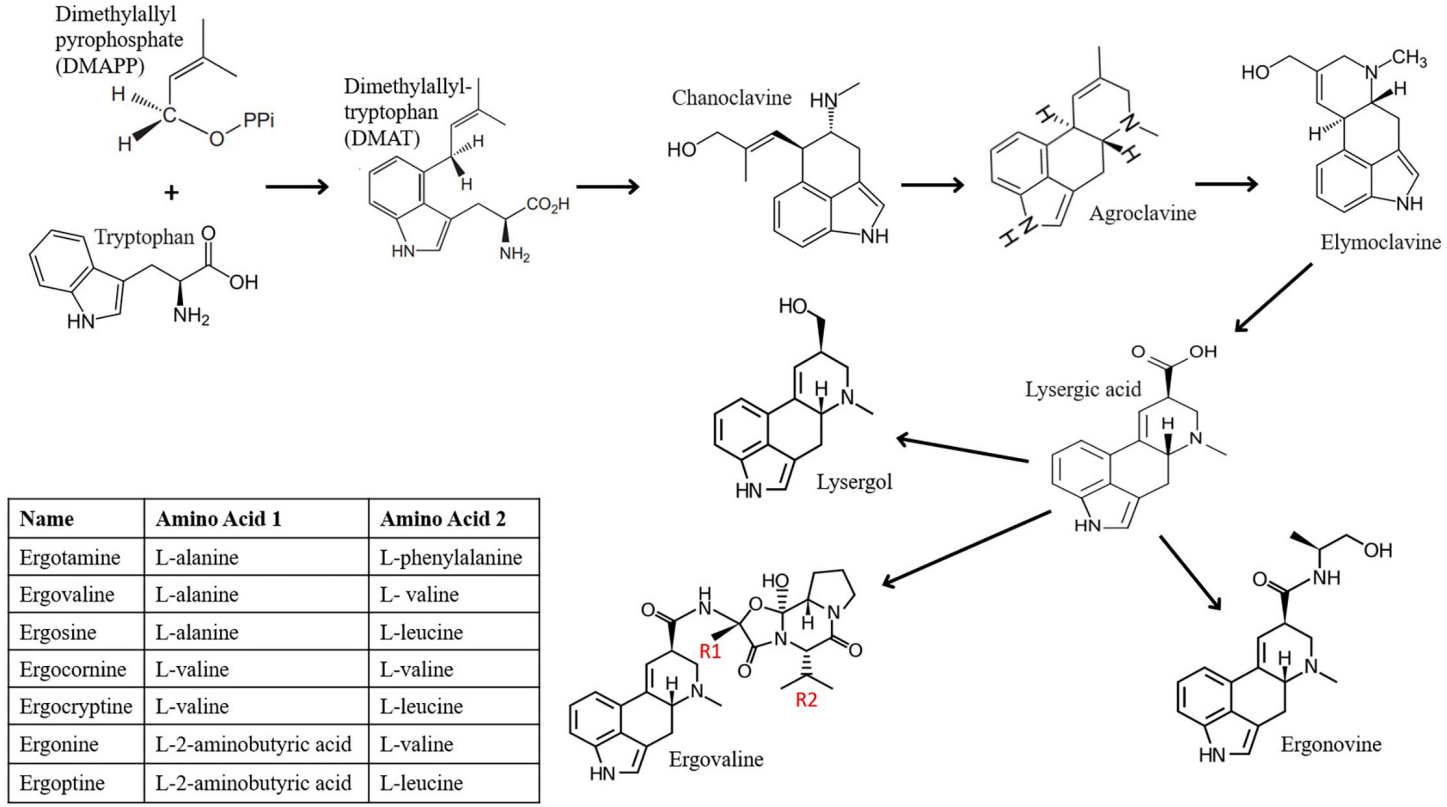
Ergot alkaloids produced by endophytic fungi of the genus Epichloë in infected plants.

Biosynthesis of ergot alkaloids (Figure 1) was first studied in *Claviceps purpurea*, the fungus responsible for ergotism and initial suspect for the cause of fescue toxicosis. The precursors of ergot alkaloids are tryptophan and the mevalonic acid derivative dimethylallyl pyrophosphate (DMAPP), which originates from acetate metabolism (64, 65). These are converted to dimethylallyl-tryptophan (DMAT) by DMAT synthase, in what is probably the limiting step in ergot alkaloid formation (64). N-methylation and C-oxidation of DMAT leads to formation of a diene, which is then epoxidized, resulting in spontaneous cyclization of the C-ring and release of an α-carboxyl group, yielding chanoclavine I; the first of the clavines (66). The cyclization of the D-ring of chanoclavine I is catalyzed sequentially by chanoclavine I dehydrogenase, a flavine-dependent oxidoreductase, then a reductase, to produce the tetracyclic clavine alkaloid, agroclavine (67). Agroclavine is transformed into lysergic acid by the NADPH-dependent cytochrome P450, CloA, which is responsible for three rounds of 2-electron oxidations (67). The only difference between the clavines and the derivatives of lysergic acid are the isomerization of the double bond in the D ring of agroclavine from the 8, 9 to the 9, 10 position when the lysergic moiety is formed (68).

A variety of amide derivatives of lysergic acid have been characterized in endophyte-infected plants; the simplest being ergolines and the most complex being ergopeptines. Ergoline alkaloids have a simple chemical structure attached to the 8 carbon; lysergic acid has a carboxylic acid and lysergol has a methyl alcohol in that position, while others may include hydrazide, azide, or amide groups (69). Ergopeptine alkaloids are created when a tricyclic peptide ring is attached *via* a carbonyl at the 8 position in the D ring of lysergic acid (68). Three amino acids form a tricyclic peptide, proline always occupies position 3, while the amino acids in positions 1 and 2 will differ depending on the alkaloid formed (63). Biosynthesis of ergopeptide lactams, the immediate precursors of ergopeptines, are catalyzed by the non-ribosomal peptide synthetase (NRPS) enzyme, lysergyl peptide synthetase (LPS), which consists of two subunits (67). The subunit LpsB, has a single module that activates D-lysergic acid, and the subunit LpsA has three modules that each specify a hydrophobic L-amino acid or an analog; once these four substituents are linked, they are released from the NRPS and the amino acids in position 2 and 3 are cyclized to form the piperizinedione ring (67). EasH, a dioxygenase, catalyzes the oxidative cyclization of the amino acid in position 1, forming the final cyclol ring of an ergopeptine (67). Condensation of D-lysergic acid with alanine, valine, and proline for example, forms ergovaline, which as mentioned previously, is the most prevalent ergopeptine in E+ tall fescue. The R_1_ position can be occupied by alanine, valine or L-2-aminobutyric acid and the R_2_ position can hold valine, isoleucine, leucine, or phenyalanine (70), in order to produce other ergopeptines in E+ fescue, which include ergotamine, ergosine, ergocornine, ergocristine, ergokryptine, ergonine (46).

### Absorption

Alkaloids must overcome several protective mechanisms the body has in place before they cause disruption in the physiological systems of an animal. These natural barriers include mechanisms of influx and efflux, biotransformation, impediments during transport such as the blood-brain and placental barriers, and physical elimination from the body (23). *In vivo* evidence of ergot alkaloid absorption has relied on analysis of excretions and intake estimates in the live animal. Studies using radiolabeled ergot alkaloids in isotopic experiments in rats, dogs, and monkeys, found that the route of excretion of the ergot alkaloids are dependent on the molecular weight and polarity of the compound (71). Alkaloids with a molecular weight of <350 Da (lysergic acid and its amides) are excreted in the urine, those between 350 and 450 Da are excreted in urine and bile, and those above 450 Da (ergopeptines) are excreted in the bile and eliminated through feces (71). Samples of urine and bile taken from cattle grazing endophyte infested tall fescue demonstrated that 96% of the ergot alkaloids consumed will be excreted in the urine as lysergic acid derivatives and very little in the bile as ergopeptines (72). The urine collected contained most of the ergot alkaloids consumed, suggesting that the excretory form of ergot alkaloids is either intact lysergic acid derivatives or biotransformed ergopeptine alkaloids. Ergopeptines are the major ergot alkaloid in tall fescue, not lysergic acid derivatives, so it is unlikely that the concentration in the forage is high enough to account for the differences between urinary and biliary excretion patterns (72). Recovery of ergovaline was determined using samples taken from the abomasum and feces of sheep fed E+ and E- fescue seed; 47–63% was recovered in abomasal digesta, indicating a significant reduction in the foregut, but only 6–7% was recovered from the feces, indicating most of the remaining alkaloids were absorbed or metabolized in the intestines (73). Ergopeptines analyzed *via* HPLC in the feces of ergot-exposed dairy cattle showed that almost 24% of their ergopeptine intake was excreted in the feces (74). A variety of factors could have contributed to the greater fecal recovery of ergopeptines by Schumann et al. (74), such as digesta flow rates, rumen pH, and differing alkaloid profiles. These *in vivo* studies supported the conclusion that extensive absorption of ergot alkaloids from the gastrointestinal tract occurs in ruminants.

While many toxins and drugs are absorbed across the gastrointestinal epithelia through passive transport, the rate and magnitude of ergot alkaloid absorption is greatly affected by their solubility within the digestive tract and the extent of ionization, which in turn determines the partitioning of alkaloids between water and lipid phases (71). Most of the ergot alkaloids are weak bases and possess both polar and non-polar components, so their water/lipid partitioning and absorption are affected by the pH of the surrounding environment (23). Since ergopeptines are charged at low pH, it's assumed that they would not be absorbed in the acidic environment of the abomasum and gastric stomach, meaning absorption is confined to the forestomach, small intestine, and large intestine in ruminants, and the small intestine and large intestine in non-ruminants (23). The rumen of a forage fed animal is functionally more like the intestine than the gastric stomach. Ruminal pH is near neutral and the compartment lacks a mucosal layer, which is necessary in the gastric stomach to protect the lining from digesta. This permits nutrient transport across the epithelium of the rumen and the small intestine, making them the likely site of alkaloid absorption.

Measuring absorption across the epithelia has proven difficult and has not been done directly *in vivo*, but *in vitro* studies have provided indirect evidence to support this theory. Ruminal, reticular, and omasal tissue from endophyte naïve sheep were used in parabiotic chambers by Hill et al. to determine absorption potential for several ergot alkaloids across forestomach tissue (75). Equimolar proportions of ergot alkaloids were added to the mucosal side of the tissues and the amount of alkaloid that appeared over a 4 h period on the serosal side was measured. The rumen appeared capable of transporting ~25% more alkaloids than the omasum and ~600% more than the reticular tissues. The results also suggested that the ergoline alkaloids—lysergic acid, lysergol and ergonovine—were more likely to cross digestive barriers than ergopeptine alkaloids, specifically ergotamine and ergocryptine. Lysergic acid was transported at the highest rate in all tissues. This movement appeared to be through active transport because alkaloid transportation was reduced in the presence of sodium azide, which killed the tissue (75). These findings were supported by Ayers et al. who again demonstrated that the primary alkaloid transported across forestomach tissues is lysergic acid and additionally found that ergovaline was not transported across ruminal or omasal tissues (76). This suggested that the small intestine may be the most important site for ergopeptine absorption in ruminants. However, studies examining the role of the ruminant hindgut in alkaloid degradation and absorption have not been conducted, making it difficult to definitively determine the path of ergot alkaloids through the body.

Three potential routes were proposed by Klotz and Nicol (77) for intact ergopeptines to take once they reach the small intestine. The first is that they go unabsorbed through the small intestine and continue to the large intestine, where they face possible metabolism by microbes in the hindgut but will eventually be excreted in the feces. The second is that ergopeptines are absorbed and transported *via* the mesenteric veins to the portal vein and the liver, where they undergo hepatic detoxification or excretion back into the intestines as bile. The alternative path is that ergopeptines are absorbed in the small intestine and transported *via* the lymphatic system, through the thoracic duct and into venous circulation at the subclavian vein; this would bypass first-pass detoxification by the liver and allow ergopeptines to be systemically distributed *via* arterial blood, before detoxification by the liver. Considering lipids are regularly absorbed from the small intestine through lacteals leading to the lymphatic system in the form of micelles, and ergopeptines are lipophilic in nature, this route of absorption is likely to occur in ruminants (77).

### Metabolism

Ergot alkaloid metabolism in ruminants cannot be fully explained without considering the role played by the microorganisms responsible for pre-gastric fermentation of feedstuffs. The diverse microbial community of the rumen can catabolize substrates, like cellulose, and liberate compounds that are not otherwise accessible for mammalian digestion. Microbial enzymes exist that are capable of metabolizing or modifying a wide array of plant primary and secondary metabolites, including toxicants. *In vitro* and *in vivo* fermentation studies have been used to elucidate the actions of microbes on fescue digestion and alkaloid metabolism in the rumen. When endophyte infested fescue was incubated with viable microbes *in vitro*, the concentration of soluble ergovaline decreased linearly as fermentation progressed, with only 5% remaining after 48 h of incubation (78). The total amount of ergot alkaloids has been found to increase over time, with lysergic acid being the primary alkaloid in both *in vitro* and *in vivo* ruminal fluids (76). The digestion of ergovaline and production of lysergic acid has been investigated *in vivo* by De Lorme et al. as well (79). Lambs were fed endophyte infested hay, and urine and feces were collected 21 and 25 days later. Samples were analyzed for ergot alkaloids using HPLC. Fecal and urinary excretion of the compounds indicated that lysergic acid was produced during digestion of fescue, most likely in the rumen, which previous research has shown is a likely place of absorption (79). These actions by microbes in the rumen increase the concentrations of ergot alkaloids available for absorption by the animal's gastrointestinal tract and subsequently increase the chance of intoxication from fescue consumption.

Alkaloids absorbed from the rumen and across the epithelium of the small intestine enter the blood stream, where they are quickly removed from circulation by the liver and eliminated from the body. Research into the rate of clearance of these compounds for livestock has been limited, but it has been demonstrated in sheep that ergovaline injected intravenously disappeared from the blood within 1 h of administration (80). It should also be noted that lysergic acid in particular, begins to appear in the urine of steers that have been moved from E- fescue pasture to E+ pasture within 24 h, demonstrating the potential speed of alkaloid metabolism and elimination in ruminants (72). Pharmacokinetic studies conducted in non-ruminant species using bromocriptine have found that extensive first-pass biotransformation of this peptide occurred in the liver (81). The liver possibly provides additional alteration and degradation of ergot alkaloids in ruminants but there is little information concerning that route of metabolism in livestock available. In light of this, it is assumed that post-ruminal biotransformation of ergot alkaloids in ruminants is similar to that reported in laboratory species and humans.

Primary oxidation of a wide variety of steroids, fatty acids, and prostaglandins, as well as drugs and environmental pollutants in the liver is catalyzed by the superfamily of cytochrome P450 enzymes (82). In humans, biotransformation of ergot alkaloids is generally mediated by the CYP3A subfamily of cytochrome P450, which consists of 3 genes: CYP3A4, CYP3A5, and CYP3A7 (83). CYP3A4 is the predominant P450 enzyme in the human liver and intestine and has a very active role in the metabolism of drugs and toxicants (23). The ergopeptines, bromocriptine, ergocryptine, and dihydroergotamine, were found to strongly interact with rat liver microsomes by Peyronneau et al. as a result of the ergopeptines binding to a protein site close to the heme (82). They also demonstrated that the tripeptide portion of ergopeptines was essential for these alkaloids to be recognized and bound to CYP3A, and alkaloids that lack the moiety, such as lysergic acid derivatives, failed to bind with CYP3A. Ergotamine has been shown to undergo enzymatic modification by CYP3A in cattle microsomes as well, being converted to more hydrophilic metabolites called M1 and M2 (8-hydroxy derivatives), which then undergo a second hydroxylation to become M3 and M4 (8, 9-dihydroxy derivatives) (84). Little is known about these resulting metabolites, their effects on the body or the role they play in fescue toxicosis. Further research is needed to illuminate the process undergone by ergot alkaloids once they reach the liver.

### Downstream Effects on Receptors

As previously mentioned, consumption of endophyte infected tall fescue and subsequent liberation of ergot alkaloids into an animal's gastrointestinal tract can cause a variety of physical effects down the line, such as fescue foot, summer slump, and fat necrosis. These diverse biological effects are directly related to the structural similarities between the tetracyclic ergoline ring of ergot alkaloids and the biogenic amine neurotransmitters norepinephrine, dopamine, and serotonin (70). The biogenic amides are associated with transmembrane G protein-coupled receptors which are located throughout the body in varying populations depending on tissue type (85). As ligands, ergot alkaloids can stimulate these receptors by acting as agonists or partial agonists or they can stifle them by acting as antagonists (86). To add to the complexity of the issue, serotoninergic, dopaminergic and adrenergic receptors are not homogenous. At least 14 distinct subtypes of serotonin receptors, 5 subtypes of dopamine receptors, and at least 10 subtypes of adrenoceptors have been identified based on structural, transductional, operational, and functional differences (86). Additionally, slight structural differences between alkaloids can vary their specificity to receptor systems and selectivity among the various subtypes (87, 88). The non-competitive antagonism created by ergot alkaloids binding to these receptors prevents them from performing correctly. Persistent binding of alkaloids results in receptor desensitization from prolonged stimulation, in turn prompting reduced signal transduction (89). Moreover, agonist occupancy has been shown to enhance the rate of receptor internalization (90) and excessive binding creates an opportunity for alkaloids to accumulate in tissues, which may be released during tissue turnover (85). Continuous consumption of toxic fescue and sustained exposure to ergot alkaloids can have huge repercussions on biological processes if the rate of alkaloid accumulation on receptors exceeds the rate of receptor turnover. Animals exposed to ergot alkaloids exhibit a diverse and variable set of symptoms which depend on the type and location of the receptors affected, the quantity and structure of the alkaloid, and any environmental stressors that accompany consumption.

### Serotonin Receptors

Serotonin or 5-hydroxytryptamine (5-HT) receptors are located throughout the mammalian body in the central and peripheral nervous system, and in blood vessels, the gastrointestinal tract and on platelets (81). They are responsible for modulating the release of many neurotransmitters and hormones, which influence biological processes like aggression, appetite, mood, nausea and thermoregulation. The first demonstration of ergot alkaloids directly acting on 5-HT receptors in bovine vasculature was from Dyer (47), who exposed bovine uterine and umbilical arteries to ergovaline, and showed the alkaloid elicited a contraction of both arteries. Additionally, they significantly antagonized contractions to ergovaline using ketanserin, a 5-HT_2_ receptor antagonist, confirming the contraction of vascular smooth muscle was associated with activation of 5-HT_2_. Later reports showed that the response of lateral saphenous veins to 5-HT_2A_ and 5-HT_7_ agonists were altered in cattle that grazed endophyte infected tall fescue (91). A series of *in vitro* studies investigated the constrictive potency of selected ergot alkaloids. They found that ergovaline was the most potent alkaloid, followed by ergotamine with a very similar contractile dose response curve to ergovaline, then intermediate responses from ergocryptine, ergocristine, ergocornine, and ergonovine, and lastly the least potent was lysergic acid (87, 92–94). 5-HT_2_ receptors have also been found in lung tissue and have been shown to be involved with pulmonary vasoconstriction and bronchoconstriction in cattle (81). This may help explain the pronounced breathing difficulties experienced by cattle on E+ pasture, especially during the summer when animals are exposed to high heat situations. While vasoconstriction has been the focus for much of the serotonergic receptor research, the effects of ergot alkaloids on the variety of 5-HT receptors in the body have not been fully examined. Increased serotonin is known to suppress appetite by acting on the hypothalamic satiety center (95), and it is involved in the regulation of gastrointestinal motility (96). These are possible routes for ergot alkaloids to reduce feed intake and negatively affect motility and passage rate.

### Adrenergic Receptors

Adrenergic receptors are located on blood vessels throughout the body and generally stimulate the sympathetic nervous system, which is responsible for the fight-or-flight response. When these receptors are triggered, heart rate and blood pressure increase, and blood flow is diverted from non-essential organs to skeletal muscle. Cattle receiving endophyte-infected fescue hay or seed have shown large variations in plasma concentrations of norepinephrine and epinephrine, which resulted in nervous and highly excitable animals (39). Disruption of adrenergic receptors occurs quickly after consumption of endophyte infected fescue and causes persistent vasoconstriction and dysfunction of blood vessels in the extremities. Heifers showed reduced blood flow and narrowing of the lumen of the caudal artery just 4 h after eating E+ fescue (97). This can result in thickening and damage to the vessel's endothelial lining, as well as edema and thrombosis (98). Tissue necrosis or dry gangrene is a direct result and a severe consequence of endophyte consumption.

There are two groups of adrenoreceptors, α (subtypes: α_1_ and α_2_) and β (subtypes: β_1_, β_2_, and β_3_). There is no evidence that ergot alkaloids either stimulate or block β adrenoreceptors (99). Both α_1_ and α_2_ adrenergic receptors have been found on the dorsal pedal vein of cattle (100). Lateral saphenous veins from cattle grazing E+ and E- pastures showed no difference in contractile response when exposed to phenylephrine, an α_1_ receptor agonist, demonstrating that α_1_ receptors are not affected by ergot alkaloid consumption. Conversely, when veins from E+ animals were exposed to BHT-920, an α_2_ receptor agonist, they had enhanced reactivity and greater contractile response, demonstrating the involvement of α_2_ receptors in the vascular effects of ergot alkaloids (101). E+ fescue consumption has been found to decrease blood flow to core and peripheral tissues, including rib and leg skin, adrenal glands, cerebellum, duodenum, and colon (102). These types of changes were expected to be accompanied by increased blood flow to other areas but the blood flow to other tissues remained unchanged. This led to the conclusion that E+ fescue reduced the cardiac output in cattle (102). During continuous heat challenge, animals consuming E+ fescue experienced a shift in core body temperature from 38.8 to 39.2°C; this was accompanied by increases in respiration rate, respiratory vaporization, skin vaporization, and skin temperature but not a significant change in heat production (103). Researchers believe that vasoconstriction mediated by α-adrenergic receptors in subcutaneous areas reduces evaporative heat loss from the skin in heat stress situations, thus causing the increased core temperature seen in cattle afflicted with fescue toxicosis. α_2_ receptors are also present on blood platelets, and when they are stimulated by ergot alkaloids platelet production of thromboxane increases, which in turn induces platelet aggregation and arterial constriction (81). This combined with bronchial constriction caused by alkaloid influence on serotonergic receptors, results in reduced blood O_2_ saturation and tissue oxygenation. This can explain the increased respiration rate seen by Al-Haidary et al. (103).

### Dopamine Receptors

The five subtypes of dopamine receptors are divided into D1-like (D1 and D5) and D2-like (D2, D3, and D4) subfamilies. D1 and D2 receptor subtypes are located in the neostriatum of the brain, where dopamine is important for motor function. D2 receptors are also found at high levels in the pituitary gland. D3 receptors are in the limbic regions of the brain, where they control aspects of behavior, emotion, motivation, and cognition. D4 receptors are sometimes found in the brain but are more common in the cardiovascular system (86). Ergot alkaloids are agonistic to D2 receptors, which results in a significant reduction in both circulating and releasable prolactin from the anterior pituitary, a classic indication of fescue toxicosis (104). Prolactin is essential for regulating metabolism, the immune system, and pancreatic development, as well as regulating milk synthesis and secretion. Decreased milk production in cows and ewes, and agalactia in mares grazing E+ pasture is a result of the decline in circulating prolactin (105). At the onset of spring, longer days typically elevate prolactin levels to initiate shedding of the winter hair coat, but animals suffering from ergot alkaloid poisoning have prolactin levels that are too low to trigger shedding. These animals will maintain a shaggy coat, even in the heat of summer, which further exacerbates heat stress and elevates core body temperature triggered by alkaloid-induced vasoconstriction (106). Researchers have suggested that decreased prolactin levels are also involved in the reduced reproduction seen in seasonal breeding livestock species grazing E+ fescue pastures (23). Intravenous administration of ergotamine to cattle has resulted in decreased plasma insulin concentrations and increased glucagon within 1 h of dosing (107). The exact mechanism for these effects is still unknown. It has been shown that D2 receptors may be involved in pancreatic function and associated insulin regulation (108). Disruption of these dopamine receptors by agonists has resulted in decreased insulin response and glucose intolerance (109). More research in this area could provide the mechanism by which ergot alkaloids affect insulin secretion in cattle.

## Pathogenesis of Fescue Toxicosis

The majority of ergot alkaloid research in the past 50 years has focused on observing phenotypic changes that occur in livestock when they consume tall fescue (Table 1). Recent forays into the metabolome, transcriptome, and proteome of livestock affected by ergot alkaloids have demonstrated that the pathogenesis of fescue toxicity-related illnesses are much more complex than previous research suggests. Metabolomics is an exciting area of study which uses the comprehensive measurement of metabolites to expose the biological pathways underlying disease etiology (120). Identification of specific metabolites pertaining to fescue toxicosis could ultimately provide biomarkers for early detection of toxicity issues in livestock, as well as new strategies for intervention and prevention of production losses. Novel research has utilized untargeted high-resolution metabolomics to analyze E+ pasture grazing-induced plasma and urine metabolome changes. Alongside ergot alkaloid metabolite detection, this method has found that tryptophan and lipid metabolism disruption were among the main consequences of endophyte consumption (117). Tryptophan metabolism through serotonin was affected by E+ consumption in opposite directions in the plasma and urine. AFMK, the product of the reaction between melatonin and reactive oxygen species (121, 122), was increased in the urine and decreased in the plasma of steers grazing E+ pastures, which may indicate early compensatory utilization of melatonin for the mitigation of ergot alkaloid-induced oxidative stress (117). Recently, it has been suggested that accumulation of ergot alkaloids in adipose tissue could be responsible for the likely disruption of lipid metabolism due to grazing E+ fescue (85). Metabolomics have also identified multiple plasma glycerophospholipids, such as phosphatidylinositol and 3-beta-D-galactosyl-*sn*-glycerol, that are affected by ergot alkaloids (117). These increased plasma glycerol metabolites could indicate disturbed glycerol utilization, which is an important energy source in ruminants (123).

**Table 1:** Fescue induced metabolic perturbations in cattle.

**Level**	**Item**	**Response**	**Citation**
Animal	Weight gain	↓	(19, 38, 39)
	Feed intake	↓	(19, 38, 39)
	Salivation	↑	(19, 110)
	Nervousness	↑	(39)
	Respiration rate	↑	(11, 39, 40, 103)
	Body temperature	↑	(11, 40, 103)
	Gangrene of the extremities	Present	(11–13, 15, 25)
	Rough hair coat	Present	(15, 40)
	Milk production	↓	(9, 21)
	Reproductive fitness	↓	(16, 111)
Tissue	Circulating cholesterol	↓	(110–113)
	Circulating insulin	↓	(107)
	Circulating IGF-1	↓	(114)
	Insulin and glucose clearance	↓	(115)
	Volatile fatty acid absorption	↓	(116)
	Vasoconstriction	↑	(11, 25, 47, 81, 93)
	Bronchoconstriction	↑	(81)
	Platelet aggregation	↑	(81)
	Thrombosis	↑	(11, 25)
	Prolactin secretion	↓	(41, 47, 104)
	Fat necrosis	↑	(15–17)
	Alkaloid accumulation	↑	(85)
Cellular	5-HT Serotonin receptor activation	↑	(47, 81, 86, 91)
	α-Adrenergic receptor activation	↑	(81, 86, 101)
	D2 Dopamine receptor activation	↑	(81, 86, 104)
	Rate of receptor internalization	↑	(90)
Metabolome	Tryptophan metabolism	Disrupted	(117)
	Lipid metabolism	Disrupted	(117)
	Plasma glycerol metabolites	↑	(117)
Transcriptome	Immune response molecules	↓	(118, 119)
	Molecular transport	Disrupted	(118, 119)
	Lipid metabolism	↓	(118, 119)
	Fatty acid metabolism	↓	(118, 119)
	Transporters	↓	(118)
	Ion channels	↓	(118)
	Uptake of carbohydrates and monosaccharides	↑	(119)
Proteome (intake restricted)	mTOR protein	↑	(115)
	S6K1 protein	No change	(115)
	4E-BP1 protein	No change	(115)

The biomarkers found from the application of metabolomics complement those from transcriptomics, which studies the transcriptome, or the complete set of RNA transcripts produced by an animal's genome under certain circumstances. It has been proposed that ergot alkaloids can alter lipid metabolism, adipose composition, and increase the occurrence of necrotic fat deposits (124), but necrotic fat has only been reported in abdominal fat deposits (125). RNA sequencing analysis from steers treated with the synthetic alkaloid bromocriptine has revealed 20 differentially expressed genes (DEG) within the mesenteric adipose and two within the intestinal epithelium (118). Changes within the intestinal epithelium included upregulation of genes indicated in increased lymphocyte trafficking and homing, which may suggest increased immune activity, and downregulation of genes indicated in transport and metabolism of long chain fatty acids and overall lipid metabolism (118). Of the DEGs found in the mesenteric adipose, 14 were downregulated genes whose functions include enzymes, transporters, ion channels, cytokines, and other immune response molecules; and six were up regulated genes with identified functions of enzyme activity, molecular transport, transmembrane receptors, cytokines, and other unknown functions (118). None of the DEGs identified had a direct effect on vascularity, but the direct involvement of some genes in immune response suggests that abdominal effects, such as necrotic fat, could be a result of persistent activation of the immune system. Of particular note, the serotonin degradation pathway in the adipose was also depressed by bromocriptine treatment, which is consistent with the previously discussed antagonistic interaction between alkaloids and serotonin metabolism. RNA sequencing has also been performed on mammary tissues from Holstein cows to identify changes in the transcriptome and molecular pathways in response to ergot alkaloids. During the initial lactation, both cattle treated with bromocriptine injections and those fed endophyte-infected seed showed enhancement in pathways involved with uptake of carbohydrates and monosaccharides, while activities such as fatty acid metabolism, molecular transport, and lipid conversion decreased (119). During the subsequent lactation, when there was a negative energy balance, these treatments increased gluconeogenesis and decreased pathways for other metabolic activities such as fatty acid metabolism and oxidation of lipids, as well as development of the mammary gland (119). These changes in the mammary gland transcriptome were consistent with the observed decrease in milk production seen in cattle exposed to endophyte-infected fescue. Animals exposed during the dry period did not have reduced milk yield in the following lactation after consumption ended (126). However, cattle treated with bromocriptine and endophyte-infected seed showed decreases in pathways involving leukocyte and lymphocyte function (119). This could be relevant to animal health because the mammary gland is susceptible to mastitis during the dry period, and lost function in these pathways may illustrate the downside to grazing dairy cattle on endophyte-infected fescue during the dry period (119). RNA sequencing data provides insight into specific tissue responses and potential pathways and genes affected by ergot alkaloid consumption, possibly leading to the discovery of the mechanisms driving declining health in production livestock.

Another useful tool in the search for specific biomarkers for fescue-related illnesses is proteomics. Mapping the entirety of proteins produced and modified in cattle, their structure and function, as well as the complex protein-protein interactions in the bovine system is integral for developing disease treatments and effective diagnostic tools. Of particular importance to fescue toxicity research is the mTOR pathway and its multitude of related proteins. The mTOR signaling pathway holds a central position in regulating cell growth and metabolism through mediation by two multi-protein complexes, mTORC1 and mTORC2 (127). For growing cattle, proteins involved with mTORC1, along with its upstream and downstream relations, provide the central control of nutrient and energy sensing, making them essential for maintaining the balance between anabolism and catabolism in response to environmental conditions. Western blotting has been utilized to study the mTOR pathway and protein synthesis in the muscle of growing steers treated with bromocriptine, which was used to induce a fescue toxicosis-like syndrome. Bromocriptine decreased insulin and glucose clearance, indicating decreased insulin sensitivity (115). The primary action of insulin in skeletal muscle is to stimulate glucose uptake and metabolism through activation of insulin receptors (IR) on the surface of muscle cells. Thus, ergot alkaloid perturbation of insulin signaling could impact downstream activation of mTOR pathway proteins. Although this disturbance in insulin signaling occurred, bromocriptine treatment did not affect downstream activation of S6K1 or 4E-BP1 proteins, so does not appear to inhibit activation of the mTOR pathway or protein synthesis (115). This suggests that decreased performance in cattle exposed to fescue-derived alkaloids may stem from interference with glucose homeostasis and skeletal muscle metabolism due to disruption of signals upstream of mTOR, or disturbances in mTOR independent pathways. This area of study is relatively new in cattle and there is still much to be investigated.

Research of this nature is crucial for further exploration of new treatments and diagnostic strategies for fescue toxicosis. Past solutions have focused on surface level issues such as improving weight gains in grazing animals, but there has been little work that goes further into the mechanisms responsible for the development of fescue toxicity-related illnesses. Investigation of the effects of ergot alkaloids on the metabolome, transcriptome, and proteome of livestock will be crucial for establishing a true understanding of how ergot alkaloids disturb whole-body homeostasis.

## Conclusion

The story of tall fescue is tightly woven with that of the endophyte *Epichloëë coenophiala*. Modern grazing operations cannot ignore the benefits and drawbacks of the relationship. Novel endophyte cultivars have been developed to replace toxic tall fescue, but the reality remains, that a majority of tall fescue pastures in the United States still produce toxic ergot alkaloids that cause biological issues for livestock. It is known that their structural similarities to neurotransmitters allow alkaloids to bind to receptors throughout the body and cause a variety of physiological issues in livestock. Further investigation into the absorption and disposal of ergot alkaloids by the animal's body as well as their impact on digestion and metabolic pathways are necessary to develop effective solutions for the multitude of performance-compromising effects of endophytic alkaloids.

## Author Contributions

TF prepared figures and drafted manuscript. TF, EV, and KM edited and revised manuscript and approved final version of manuscript. All authors contributed to the article and approved the submitted version.

## Funding

This work was supported by National Institute of Food and Agriculture, U.S. Department of Agriculture Multistate Program under 1018136.

## Conflict of Interest

The authors declare that the research was conducted in the absence of any commercial or financial relationships that could be construed as a potential conflict of interest.

## Publisher's Note

All claims expressed in this article are solely those of the authors and do not necessarily represent those of their affiliated organizations, or those of the publisher, the editors and the reviewers. Any product that may be evaluated in this article, or claim that may be made by its manufacturer, is not guaranteed or endorsed by the publisher.
